# A Light-Inducible Split-dCas9 System for Inhibiting the Progression of Bladder Cancer Cells by Activating p53 and E-cadherin

**DOI:** 10.3389/fmolb.2020.627848

**Published:** 2021-01-05

**Authors:** Xinbo Huang, Qun Zhou, Mingxia Wang, Congcong Cao, Qian Ma, Jing Ye, Yaoting Gui

**Affiliations:** ^1^Guangdong and Shenzhen Key Laboratory of Male Reproductive Medicine and Genetics, Institute of Urology, Peking University Shenzhen Hospital, Shenzhen-Peking University-The Hong Kong University of Science and Technology Medical Center, Shenzhen, China; ^2^Department of Urology, The Affiliated Nanhua Hospital of University of South China, Hengyang, China

**Keywords:** CRISPR, split-dCas9, light-inducible, bladder cancer, logic gate

## Abstract

Optogenetic systems have been increasingly investigated in the field of biomedicine. Previous studies had found the inhibitory effect of the light-inducible genetic circuits on cancer cell growth. In our study, we applied an AND logic gates to the light-inducible genetic circuits to inhibit the cancer cells more specifically. The circuit would only be activated in the presence of both the human telomerase reverse transcriptase (hTERT) and the human uroplakin II (hUPII) promoter. The activated logic gate led to the expression of the p53 or E-cadherin protein, which could inhibit the biological function of tumor cells. In addition, we split the dCas9 protein to reduce the size of the synthetic circuit compared to the full-length dCas9. This light-inducible system provides a potential therapeutic strategy for future bladder cancer.

## Introduction

Bladder cancer ranks as the tenth highest incidence with ~550 thousand people diagnosed worldwide in 2018. The incidence in males is significantly higher than in females, and the highest incidence is observed in men from Europe and North America (https://gco.iarc.fr/today/data/factsheets/cancers/30-Bladder-fact-sheet.pdf). Bladder cancer is often associated with a poor prognosis since the surgery naturally involves the urogenital tract, which leads to a profound psychological impact as well as the physical impact (2017). In addition to the surgery, chemotherapy is often considered as a part of the combination therapy (Leow et al., 2014). However, poor patient compliance due to the side effects and the lack of durable response evokes an urgent need for more targeted and personalized approaches (Vasekar et al., 2016).

Synthetic biology and gene therapy have enormous potential in satisfying these requirements (Rivière and Sadelain, 2017; Sedlmayer et al., 2018). After several decades of development, we now acquire various genetic editing methods, like TALEN(transcription activator-like effector nucleases) (Beerli et al., 2000), ZFNs(zinc-finger nucleases) (Zhang et al., 2011) and CRISPR (clustered regularly interspaced short palindromic repeats) technology. (Jinek et al., 2012; Qi et al., 2013). The high efficiency, ease of use and low cost soon make the CRISPR a preferable tool in the field of gene editing (Zhan et al., 2019). Small molecules have long been the preferential choice for regulating gene expression (Gossen et al., 1995; Schenone et al., 2013). They present great regulatory performance *in vitro* and *in vivo*, however, they often trigger side effects for therapeutic use (Muller and Milton, 2012). Thus, scientists proposed that the future transcription-control systems will be a molecule-free or traceless remote control (Folcher et al., 2014; Ye and Fussenegger, 2019). Following this, increasing investigation upon light-inducible gene-regulating devices was conducted during the recent decade. Other than molecule-free, light control systems allow precise spatial and temporal regulation of cell behavior, which makes it ideal for gene regulation (Polstein and Gersbach, 2015).

Previous studies found that under blue light illumination, the cryptochrome 2 (CRY2) photoreceptor could form a heterodimer with its specific binding CIB1 protein (cryptochrome-interacting basic-helix-loop-helix 1) (Yamada et al., 2020; Zhao et al., 2020). Incorporated this light-responsive module to the dCas9 protein and the transcriptional activation domain could flexibly tune the transcriptional regulatory function of dCas9 protein. However, applying the CRISPR-dCas9 circuits to clinical use is hindered by the cargo size of current viral delivery vehicles (Truong et al., 2015; Li et al., 2018). To solve this problem, researchers proposed that dCas9 protein can be split into different domains and integrated inside the cells (Nihongaki et al., 2015; Zetsche et al., 2015; Ma et al., 2016).

In this study, we established a light-induced gene expression device based on the dCas9 protein and the CRY2-CIB1 photosensitive module. Then, we incorporated a modular AND logic gate with a CRISPR-dCas9 system for improved specificity. The system would only activate the output gene in the presence of both inputs and the blue light (Liu et al., 2014). In addition, for better practicality, we reduced the size of the dCas9 protein by splitting it in half. Our results demonstrated that by activating the exogenous p53 or endogenous E-cadherin via this system, the cancer cell proliferation and invasion could be effectively inhibited *in vitro*, while apoptosis was promoted. This device not only presented a more advanced specificity and modularity, but also easier to transport, which had the potential of being a promising cancer therapeutic strategy.

## Results

### Construction of the Split CRISPR-dCas9-Based Light-Inducible System

**Figure 1:**
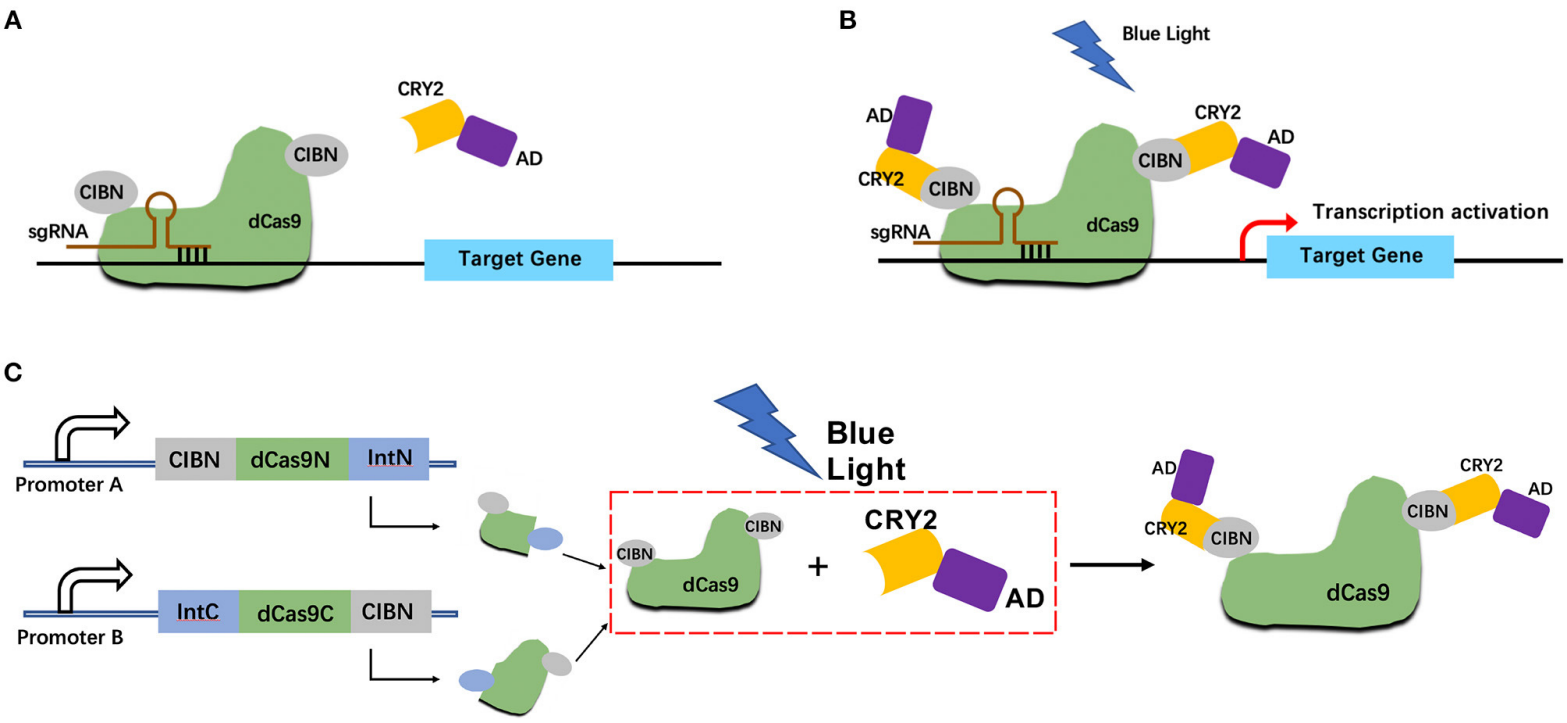
Design strategy for light-inducible split CRISPR-dCas9 gene expression system. **(A,B)** Schematic of the light-inducible CRISPR-dCas9 gene expression system without blue light illumination **(A)** and with blue light illumination **(B)**; **(C)** Schematic of the light-inducible split CRISPR-dCas9 gene expression system with blue light illumination.

We constructed a light-inducible genetic circuit that only activates the target gene expression in the presence of blue light illumination. The circuit composed of a dCas9-CIB1 fusion protein which anchored to the target gene, and a CRY2-AD (activator domain) fusion protein which acted as the transcriptional activator (Figure 1). Under the blue light condition, the CRY2 and CIBI domains were heterodimerized. Then the AD domain could activate the target gene transcription (Figure 1B). Whereas, without the blue light, the CRY2-AD component freely diffuses within the nucleus (Figure 1A).

In addition, we combined the logical AND gate with split dCas9 protein which allows the reconstitution of dCas9 only when both input promoters worked. The dCas9 protein was divided into C-terminal (IntC) and N-terminal (IntN) split inteins. The input promoter A drives the transcription of dCas9 IntN and a light-sensitive CIBN domain. The input promoter B drives the transcription of dCas9 IntC and a light-sensitive CIBN domain (Figure 1C).

### Dose-Dependent Reporter Gene Expression Induced by Blue Light Illumination

**Figure 2:**
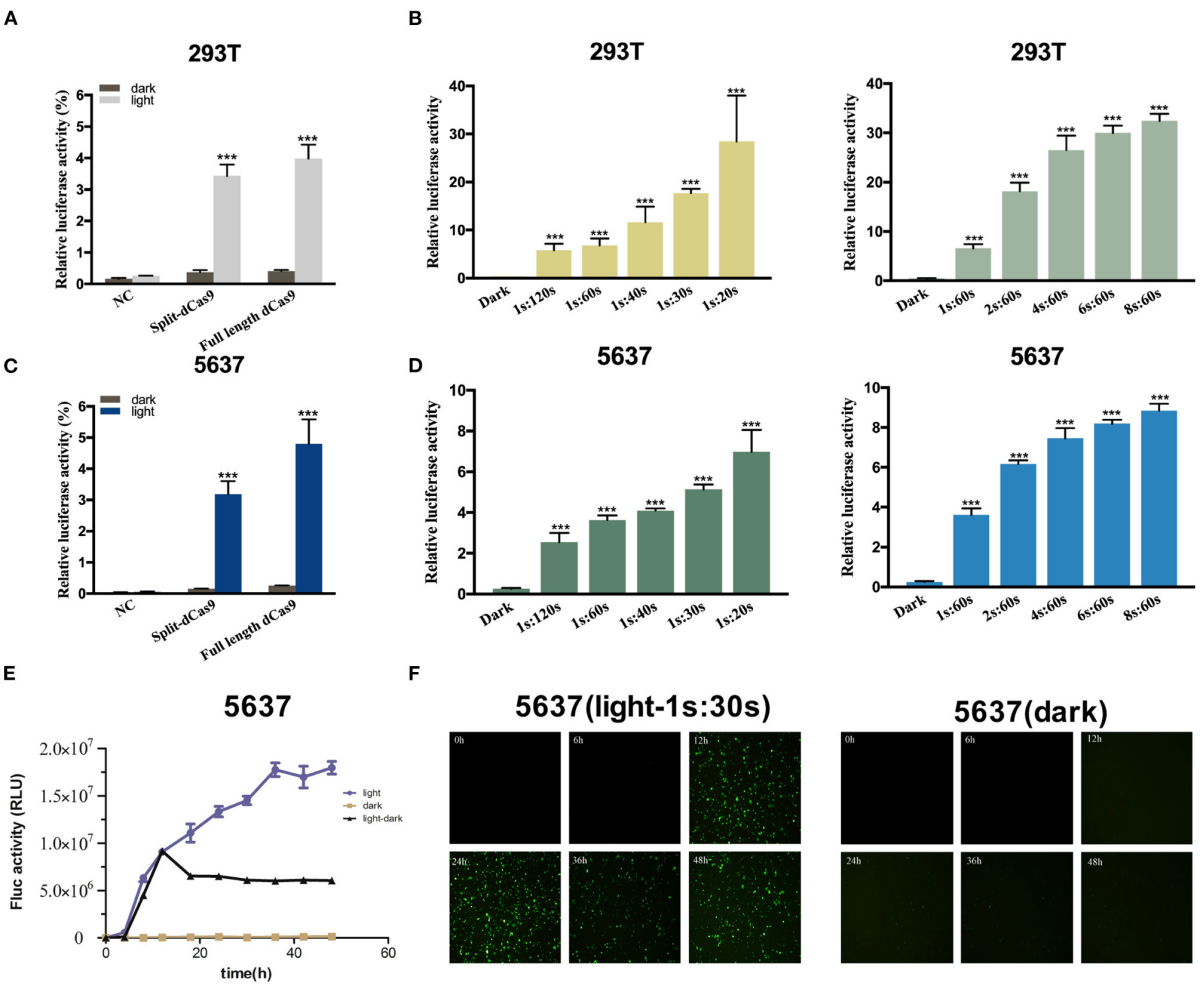
Dose-dependent luciferase activity induced by light in the cancer cell. **(A,C)** Relative luciferase activity examined of full-length dCas9 group, split dCas9 group and the negative control under dark and light condition in 293T **(A)** and 5637 **(C)** cells. **(B,D)** The relative luciferase activity increases with increasing light dose in 293T **(B)** and 5637 **(D)** [1s:20s-1s:120s represents 1 s illumination (0.84 W/m^2^) every 20–120 s]. **(E)** The EYFP reporter assay was used to detect changes in the expression of EYFP over time in 5,637 cells under light, dark, and light-dark conditions. **(F)** Time course of EYFP gene expression under light, dark, and light-dark conditions in 5,637 cell using fluorescence microscope. Data are means ± SD. (*n* = 3, *p < 0.05, **p < 0.01, ***p < 0.001).

A light-inducible CMV promoted dCas9 expression circuit was constructed to assess the efficiency of this light-inducible device. The results showed that the relative activity of hRluc luciferase production in the light group was significantly higher than that in the dark group in 293T and 5,637 cell lines (Figures 2A,C). The performance of the full-length dCas9 was better than the split dCas9. In addition, with a higher illumination dose, the relative luciferase activity increased (Figures 2B,D). Then, the time-course analysis of the expression of the fluorescence reporter gene in 5,637 cells showed that the expression level of the fluorescence gene increased with the prolongation of light (Figures 2E,F).

### Construction of the Two-Input Logic and Gate

**Figure 3:**
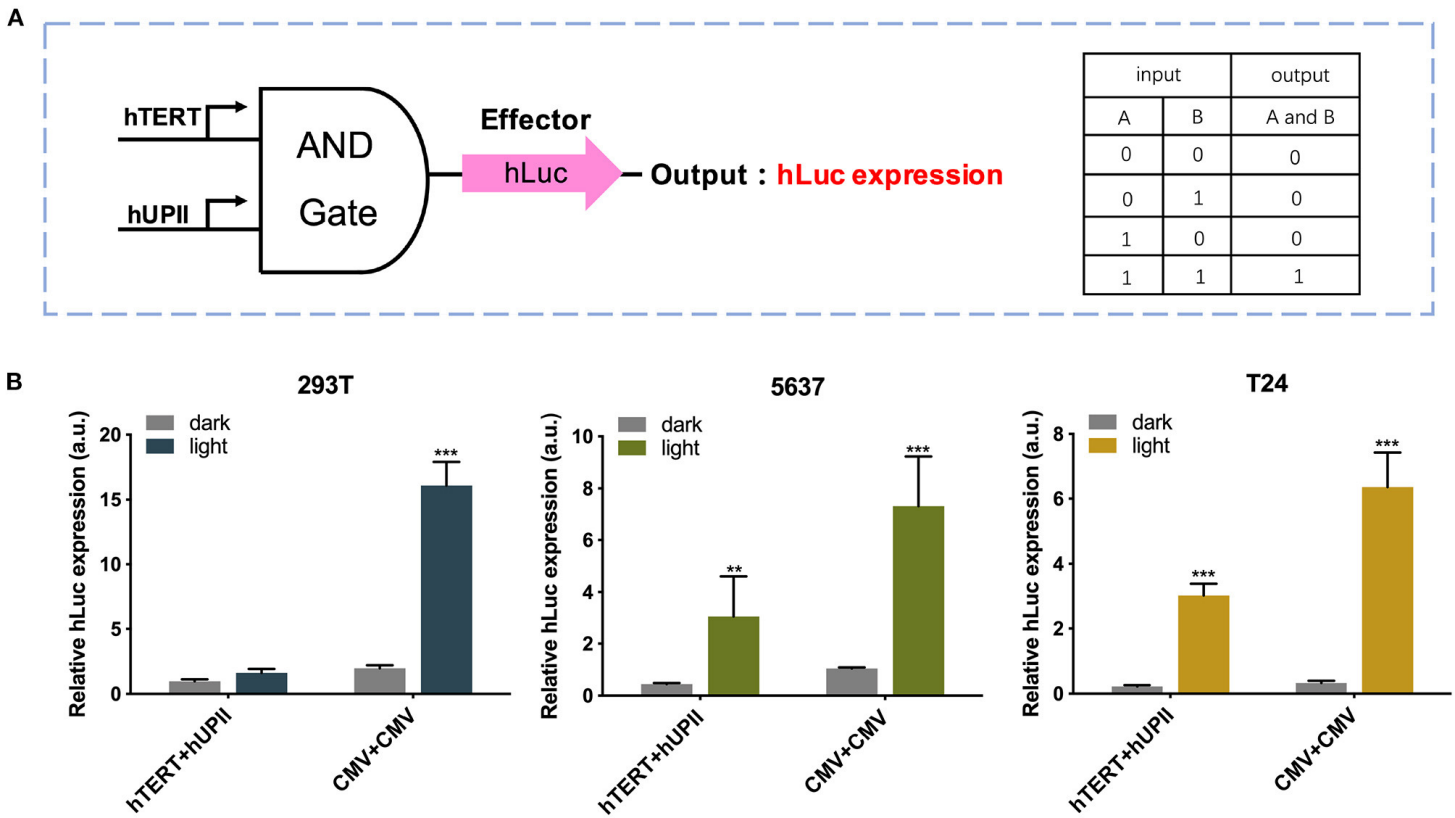
Design and validation of the AND gate genetic circuits. **(A)** The logic AND gate has the hUP II and hTERT promoters as the inputs. In the presence of both inputs, the effector hLuc expression will be activated. **(B)** Luciferase expression levels of 293T, 5,637, and T24 cell lines transfected with the hTERT/hUPII circuit under dark or blue light condition, compared to the CMV control circuit. Data are means ± SD. (*n* = 3, *p < 0.05, **p < 0.01, ***p < 0.001).

Next, we modified this system to increase its specificity toward bladder cancer cells by changing the input CMV promoter to hTERT and hUPII promoters, which are specific cancer cell and bladder cell markers (Figure 3A). The CMV circuit acted as a positive control and had higher effector expression compared to the hTERT/hUPII circuit when activated. In both 5,637 and T24 cells, both circuits were activated under light condition. Whereas, in 293T cells, only the CMV circuit was activated under light condition, no significant change was observed using the hTERT/hUPII circuit between the dark and light condition (Figure 3B).

### Construction and Optimisation of the Two-Input and Logic Circuit

**Figure 4:**
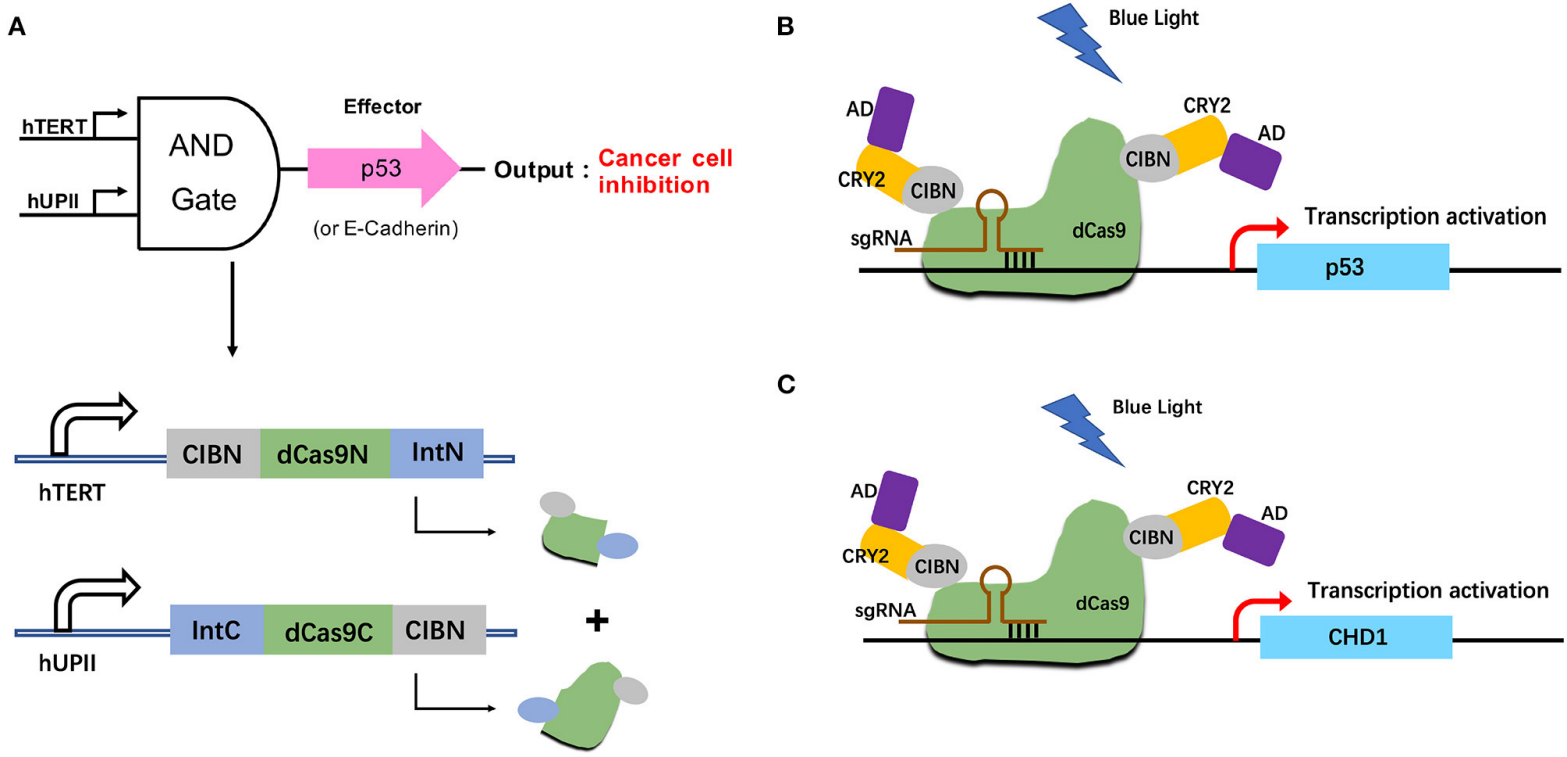
Construction of the p53/E-cadherin circuit. **(A)** The hUP II and hTERT promoters are designed to be the AND logic gate inputs, and the outputs are exogenous p53 or endogenous E-cadhenrin. **(B,C)** In the presence of both inputs and light condition, the effector p53 **(B)** or E-cadherin **(C)** gene expression will be activated.

To investigate the potential therapeutic use of this system in bladder cancer, we altered the effector to exogenous p53 and endogenous E-cadherin protein. Both of them are well-known tumor suppressors (Figure 4A). When both hTERT/hUPII promoters were activated, the dCas9-CIB1 fusion protein would be reconstituted. Under the light condition, the heterodimerized CRY2-AD and dCas9-CIBI would activate the target gene transcription under gRNA guidance. We designed gRNAs specifically targeting CHD1 gene (Figure 4C) and exogenous TRE promoter which regulate the activation of p53 expression (Figure 4B).

### The Inhibitory Effect of Dcas9-Based Light-Induced p53 Expression in Bladder Cancer Cells

**Figure 5:**
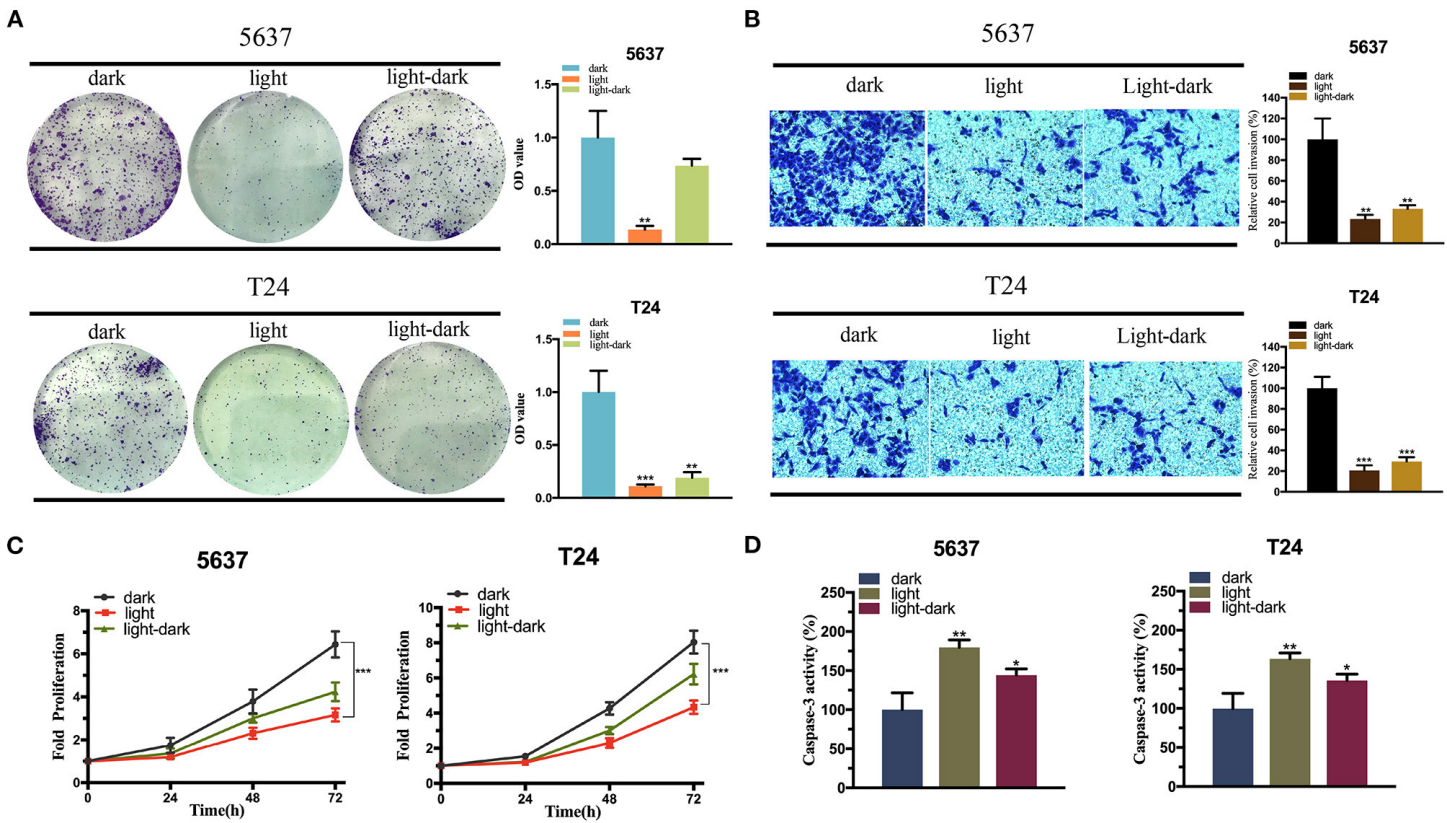
The expression of exogenous p53 inhibits the bladder cancer cell function. **(A,C)** Colony-formation assay **(A)** and CCK-8 assay **(C)** indicating the effect of light-induced exogenous p53 expression under blue-light, dark, or light-dark condition on 5,637 and T24 cell proliferation. **(B)** Transwell assay diaplaying the effect of light-induced exogenous p53 expression under blue-light, dark, or light-dark condition on 5,637 and T24 cell invasion. **(D)** Caspase-3/ELISA suggesting the effect of light-inducible exogenous p53 expression under blue-light, dark, or light-dark condition on 5,637 and T24 cell apoptosis. Data are means ± SD. (*n* = 3, *p < 0.05, **p < 0.01, ***p < 0.001).

Furthermore, we detected the expression of p53 mRNA induced by light (Supplementary Figure 1b), and examined the effect of the synthetic circuit and elevated exogenous p53 expression on the bladder cancer cells via a set of functional assays. The cell proliferation assay, CCK8 and the cell colonization assay indicated that the light-induced p53 expression significantly reduced both 5,637 and T24 cell growth in light condition (Figures 5A,C). The light-dark group significantly decreased the cell colonization in T24 cells compared to the dark control, yet in 5,637 cells, no obvious difference was observed (Figure 5A).

Then the cell invasion assay was performed. According to Figure 5B, relative cell invasion was significantly reduced in both cell lines under light condition and light-dark condition compared to the dark condition (Figure 5B). Finally, the cell apoptosis was examined using caspase-3/ELISA (enzyme-linked immunosorbent assay). The light-induced p53 expression significantly increased the cell apoptosis in both cell lines in the light group and light-dark group compared to the dark control (Figure 5D).

### The Inhibitory Effect of Dcas9-Based Light-Induced E-cadherin Circuit in Bladder Cancer Cells

**Figure 6:**
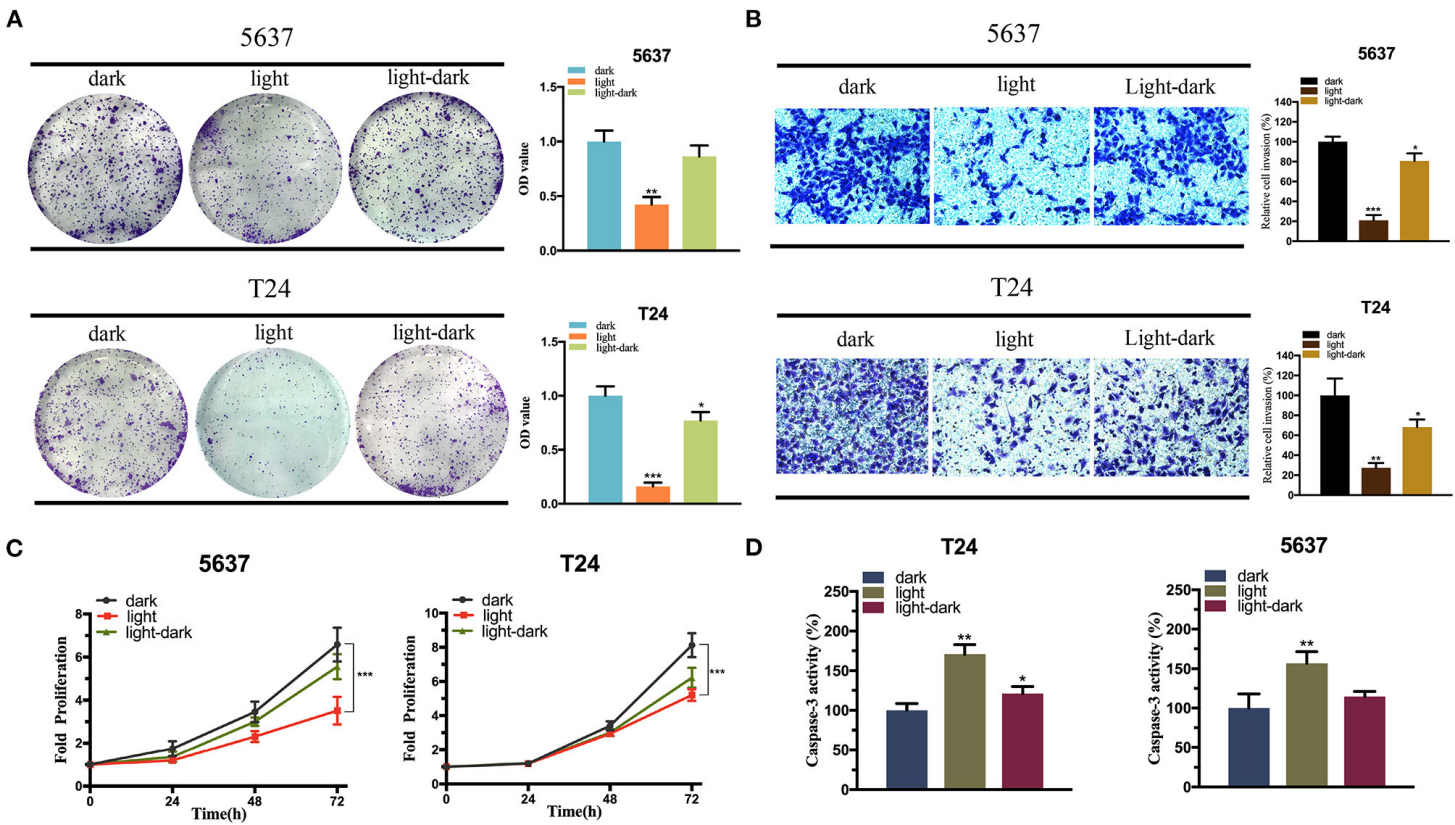
The expression of E-cadherin inhibits the bladder cancer cell function. **(A,C)** The effect of light-induced E-cadherin expression on cell proliferation examined by colony-formation assay **(A)** and CCK-8 assay **(C)** under blue-light, dark, or light-dark condition in 5,637 and T24 cells. **(B)** The effect of light-induced E-cadherin expression on cell invasion examined by Transwell assay under blue-light, dark, or light-dark condition in 5,637 and T24 cells. **(D)** The effect of light-induced E-cadherin expression on cell apoptosis examined by Caspase-3/ELISA under blue-light, dark, or light-dark condition in 5,637 and T24 cells. Data are means ± SD. (*n* = 3, *p < 0.05, **p < 0.01, ***p < 0.001).

The effect of the activated E-cadherin expression was assessed on the bladder cancer cells. In addition, to optimize the efficiency of CRISPR-dCas9 system, we selected three sgRNAs for verification. The efficiency of the sgRNAs was assessed by comparing the relative CHD1 mRNA level between the experimental and control groups, and only sgRNA3 was proved to have high efficiency (Supplementary Figure 1a). The cell proliferation and colonization assay showed that the light-induced E-cadherin expression reduced both 5,637 and T24 cell growth in light condition significantly (Figures 6A,C). The light-dark group showed a decrease in cell growth, yet not as significant as the light group.

According to Figure 6B, cell invasion was also significantly affected by the expression of light-induced E-cadherin. Even under light-dark condition, the cells exhibited a large decrease in their invasion ability (Figure 6B). Finally, caspase-3/ELISA (enzyme-linked immunosorbent assay) performed to assess the bladder cancer cell apoptosis. The light-induced E-cadherin expression significantly promoted the cell apoptosis in both cell lines. In T24 the cell apoptosis was higher in the light-dark group compared to the dark control, yet no significant difference was observed in the 5,637 cells (Figure 6D).

## Discussion

In this study, we demonstrated that in the presence of input signals, the split dCas9 can be reconstituted and carry out its transcriptional regulatory function *in vitro*. In addition, the dose-dependent reporter gene expression induced by blue light illumination suggested a successful integration of the CRY2-CIB1 light-sensitive module with the dCas9 protein. Upon optimizing the light-induced dCas9 system, we performed functional assays to examined the effect of our system on bladder cancer cell lines and profound effects were observed.

Utilization of light-inducible gene-regulating devices in synthetic biology caught researchers' attention these years due to its precise spatial and temporal control (Polstein and Gersbach, 2015; Yamada et al., 2020; Zhao et al., 2020). The previous studies showed that a CRISPR-dCas9 based light-sensitive gene expression system could regulate an exogenous p53 expression in a dose-dependent manner (Lin et al., 2016). Via controlling the p53 expression, the bladder cancer cell proliferation was successfully inhibited. Here we adopted the idea of a light-sensitive gene-regulating device, and improved it by integrating AND logic gate with split dCas9 protein. The AND logic gate was composed of two inputs: hTERT and hUPII, which are the cancer-specific promoter and bladder specific promoter, respectively (Zhu et al., 2004; Jusiak et al., 2016). The CMV promoter, a strong promoter in eukaryotes was used as a control logic gate (Figure 3) (Zarrin et al., 1999). Through the AND logic gate, we aimed to selectively identify the bladder cancer cells and trigger the following output gene expression.

Besides the AND logic gate, we used split dCas9 protein to restrict the cargo size. This is particularly appealing to future *in vivo* applications, where the adeno-associated viral vectors (AAV) are commonly used (Santiago-Ortiz and Schaffer, 2016; George et al., 2017). They are well-known for the low immunogenicity and having various serotypes suitable for a tissue-specific infection (Verdera et al., 2020). However, the packaging of Streptococcus pyogenes (SpCas9) and gRNA is challenging due to the payload capacity of AAV is limited to ~4.7 kb (Wu et al., 2010; Senís et al., 2014). In order to solve this problem, scientists proposed various ways including finding shorter and equally efficient Cas9 analogs (Ran et al., 2015). Some researchers have applied full-length Cas9 and split Cas9 to disease treatment and compared them (Hoffmann et al., 2019). Others worked on splitting the Cas9 protein and packaged each section into an individual AAV (Moreno et al., 2018). After transfecting the cell with both AAVs, the whole protein can be reconstituted (Chew et al., 2016; Ma et al., 2016). In addition, there was also a study to expand split-Cas9 into a platform for genome-engineering applications (Wright et al., 2015). In general, the AND logic circuit and split dCas9 protein system we generated could increase the specificity and reduce the size of the device, which could potentially benefit the future therapeutic use.

The output targets of the AND logic gate was exogenous p53 or endogenous E-cadherin, whose inactivation were well-known factors that contributed to cancer development (van Roy and Berx, 2008; Valente et al., 2018; Manshouri et al., 2019; Miller et al., 2020). p53 is the most frequently mutated tumor suppressor gene in cancer. Its mutation has direct association with tumor development and functions as an oncogene (Mircetic et al., 2017; Zhan et al., 2018). E-cadherin was found to be partially or even completely lost in the malignant progression of epithelial tumors (Strumane et al., 2004; Onder et al., 2008). Others also showed that E-cadherin had strong anti-invasion and anti-metastasis effects (Mendonsa et al., 2018; Wong et al., 2018). Our results were in line with previous findings, the activation of p53 and E-cadherin had a profound effect on bladder cancer cell proliferation, invasion and apoptosis.

In conclusion, our results validated the inhibitory effect of our light-induced split dCas9 system on the bladder cancer cells. In addition, based on the previous studies, we improved the specificity and practicality of this transcriptional regulatory tool by combining the light-inducible CRISPR system with the split CRISPR-dCas9 system. This work provides a potential strategy for precise and quantitative inhibition of bladder cancer cells.

## Materials and Methods

### Plasmids Construction

The anchor domain [CMV-CIBN-dCas9(1-1153)-IntN, CMV-IntN-dCas9(1154-1368)-CIBN, hTERT-CIBN-dCas9(1-1153)-IntN, hUPII-IntN-dCas9(1154-1368)-CIBN], activator domain (CRY2PHR-VPR), EYFP reporting vector driven by Tet promoter (Tet-EYFP), dual-luciferase reporter vector driven by Tet promoter (TRE-hluc-SV40-hRluc) were purchased from Syngentech Co., Ltd. (Beijing, China). The sequence of sgRNA targeting Tet promoter of TRE-p53 vector and luciferase reporter vector: TACGTTCTCTATCACTGATA. The above plasmid abbreviations were listed in Supplementary Table 1. The relative sequences were listed in Supplementary Table 2.

### Cell Lines and Cell Culture

HEK 2923T (Human embryonic kidney cell line) was purchased from the Institute of Cell Research, Chinese Academy of Sciences (Shanghai, China). 5,637 and T24 (human bladder cancer cell lines) were purchased from American Type Culture Collection (ATCC). 293T and T24 were cultured in DMEM media (Invitrogen), 5,637 was maintained in RPMI-1640 media (Invitrogen). All cells were maintained by adding 10% fetal bovine serum, 1% penicillin/streptomycin (100 U/ml penicillin and 100 μg/ml streptomycin), and cultured in an atmosphere of 37°C and 5% CO_2_.

### Cell Transfection and Illumination

The plasmids were extracted by E.Z.N.A Fastfiler Endo-free Plasmid Maxiprep kits (Omega, Norcross, USA) from *E.coli* bacteria. Cells were transfected with a mixture of plasmids with lipofectamine 3000 (Invitrogen) according to the manufacturer's protocols. Anchor domain vector [CIBN-dCas9(1-1153)-IntN, IntN-dCas9(1154-1368)-CIBN], sgRNA-activator vector and effector vector were mixed in an amount of 0.8 μg per component in a 6-well and transfected at a ratio of 1:1:1:1. After 12 h of transfection, kept the cells in dark or under an LED lamp (460 nm, average irradiance of 0.84 W/m^2^) for culture. The illumination dose depended on the frequency and intensity of light and was controlled by a timer.

### Quantitative Real-Time PCR

According to the manufacturer's protocol, RNAeasy™ RNA Isolation Kit (Beyotime Biotechnology, China) was used to isolate total RNA from cells under different illumination conditions. cDNA was synthesized using BeyoRT™ II cDNA Synthesis Kit (Beyotime Biotechnology, China). The mRNA expression was performed using SYBR Green qPCR MasterMix (Takara, Dalian, China), with *gapdh* as the control. The relative mRNA (CDH1) level was calculated by ΔΔCt method. The primers for *gapdh* and *chd1* were shown in the following sequences, with directions ranging from 5′to 3′:

*gapdh* (F): TCCCATCACCATCTTCCA

*gapdh* (R): CATCACGCCACAGTTTCC

*p53* (F): CCTCAGCATCTTATCCGAGTGG

*p53* (R): TGGATGGTGGTACAGTCAGAGC

*cdh1* (F): ACCAGAATAAAGACCAAGTGACCA

*cdh1* (R): AGCAAGAGCAGCAGAATCAGAAT

### Cell Proliferation Assay

The effect of blue light illumination on bladder cancer cells was detected by Cell Counting Kit (CCK-8). After dark and illumination treatment, cells were seeded in 96-well-plates with 2 3 × 10^3^ cells per well and pre-incubated for 12 h. At 0, 24, 48, and 72 h, replace the medium with 100 μl fresh medium containing 10 μl of CCK reagent (Transgen, China). After incubation for 1 h, the microplate reader (Bio-Rad) was used to determine the absorbance at 450 nm of the wells. At the same time, the colony formation assay was performed to detect the clones of 5,637 and T24. The related control and light-inducible vectors were packaged by lentiviruses to infect tumor cells. The infected 5,637 and T24 cells were cultured in 6 cm culture dishes at a density of 3,000 cells per well and incubated about 10 days, during which the cells were cultured in dark, light [1s:30s, 1 second light illumination (0.84 W/m^2^) every 30 s] and light-dark (after 12 h of 1s:30s light illumination, cells were cultured in dark) condition. Finally, in order to quantify the number of cells, the cells were stained with 0.1% crystal violet and imaged. Then wash the stained cells with 33% glacial acetic acid, and measure the absorbance of each sample at 550 nm with the microplate reader.

### Cell Invasion Assay

The transwell assay was used to detect the effect of dark and light conditions on cell invasion. Digest the transfected cells and inoculate 200 μl into the transwell chamber at a density of 3 × 10^5^/ml, and the chamber was pre-plated with Matrigel (Corning, USA). The chamber was placed in the 24-well-plate, with 10% FBS medium under the cell and serum-free medium in the cell, so that cells could migrate to the medium containing serum in the lower chamber. After incubation for 24 h, the cells passing through the membrane were fixed by paraformaldehyde and stained with 0.1% crystal violet (2 mg/ml). The number of cells migrated through the membrane pores was counted under an optical microscope.

### Cell Apoptosis Assay

Cells transfected with negative control vector and light-controlled vectors were inoculated on a 12-well-plate (2 × 10^5^/well) with 70–80% confluency. After 48 h, the cell apoptosis was detected by the caspase-3/ELISA (enzyme-linked immunosorbent assay) assay (Hcusabio, China). The caspase-3 enzyme is a marker for inflammation and apoptosis signaling, since it can regulate the destruction of DNA or cytoskeletal proteins. Each test was performed at least three times.

### Statistical Analysis

All statistical data was analyzed by SPSS 21.0 software for Windows (SPSS Inc. Chicago, IL, USA). Statistical analysis was conducted using Student's t-test or ANOVA and *p*<0.05 was considered statistically significant.

## Data Availability Statement

The original contributions presented in the study are included in the article/Supplementary Material, further inquiries can be directed to the corresponding author/s.

## Author Contributions

XH, QZ, and MW performed the experiments and data analysis. XH and MW prepared diagrams and wrote the manuscript. XH and QZ designed the project. YG and JY supervised the project and provided financial support. All authors contributed to the article and approved the submitted version.

## Conflict of Interest

The authors declare that the research was conducted in the absence of any commercial or financial relationships that could be construed as a potential conflict of interest.

## References

[B1] (2017). Bladder cancer: diagnosis and management of bladder cancer: © NICE (2015) Bladder cancer: diagnosis and management of bladder cancer. BJU Int. 120, 755–765. 10.1111/bju.14045 29168333

[B2] Beerli R. R. Dreier B. Barbas C. F. III. (2000). Positive and negative regulation of endogenous genes by designed transcription factors. Proc. Natl. Acad. Sci. U.S.A. 97, 1495–1500. 10.1073/pnas.040552697 10660690 PMC26462

[B3] Chew W. L. Tabebordbar M. Cheng J. K. Mali P. Wu E. Y. Ng A. H.. (2016). A multifunctional AAV-CRISPR-Cas9 and its host response. Nat. Methods 13, 868–874. 10.1038/nmeth.3993 27595405 PMC5374744

[B4] Folcher M. Oesterle S. Zwicky K. Thekkottil T. Heymoz J. Hohmann M.. (2014). Mind-controlled transgene expression by a wireless-powered optogenetic designer cell implant. Nat. Commun. 5:5392. 10.1038/ncomms6392 25386727 PMC4241983

[B5] George L. A. Sullivan S. K. Giermasz A. Rasko J. E. J. Samelson-Jones B. J. Ducore J.. (2017). Hemophilia B gene therapy with a high-specific-activity factor IX variant. N. Engl. J. Med. 377, 2215–2227. 10.1056/NEJMoa1708538 29211678 PMC6029626

[B6] Gossen M. Freundlieb S. Bender G. Müller G. Hillen W. Bujard H. (1995). Transcriptional activation by tetracyclines in mammalian cells. Science 268, 1766–1769. 10.1126/science.7792603 7792603

[B7] Hoffmann M. D. Aschenbrenner S. Grosse S. Rapti K. Domenger C. Fakhiri J.. (2019). Cell-specific CRISPR-Cas9 activation by microRNA-dependent expression of anti-CRISPR proteins. Nucleic Acids Res. 47:e75. 10.1093/nar/gkz271 30982889 PMC6648350

[B8] Jinek M. Chylinski K. Fonfara I. Hauer M. Doudna J. A. Charpentier E. (2012). A programmable dual-RNA-guided DNA endonuclease in adaptive bacterial immunity. Science 337, 816–821. 10.1126/science.1225829 22745249 PMC6286148

[B9] Jusiak B. Cleto S. Perez-Piñera P. Lu T. K. (2016). Engineering synthetic gene circuits in living cells with CRISPR technology. Trends Biotechnol. 34, 535–547. 10.1016/j.tibtech.2015.12.014 26809780

[B10] Leow J. J. Martin-Doyle W. Rajagopal P. S. Patel C. G. Anderson E. M. Rothman A. T.. (2014). Adjuvant chemotherapy for invasive bladder cancer: a 2013 updated systematic review and meta-analysis of randomized trials. Eur. Urol. 66, 42–54. 10.1016/j.eururo.2013.08.033 24018020

[B11] Li L. Hu S. Chen X. (2018). Non-viral delivery systems for CRISPR/Cas9-based genome editing: challenges and opportunities. Biomaterials 171, 207–218. 10.1016/j.biomaterials.2018.04.031 29704747 PMC5944364

[B12] Lin F. Dong L. Wang W. Liu Y. Huang W. Cai Z. (2016). An efficient light-inducible P53 expression system for inhibiting proliferation of bladder cancer cell. Int. J. Biol. Sci. 12, 1273–1278. 10.7150/ijbs.16162 27766041 PMC5069448

[B13] Liu Y. Zeng Y. Liu L. Zhuang C. Fu X. Huang W.. (2014). Synthesizing AND gate genetic circuits based on CRISPR-Cas9 for identification of bladder cancer cells. Nat. Commun. 5:5393. 10.1038/ncomms6393 25373919

[B14] Ma D. Peng S. Xie Z. (2016). Integration and exchange of split dCas9 domains for transcriptional controls in mammalian cells. Nat. Commun. 7:13056. 10.1038/ncomms13056 27694915 PMC5063958

[B15] Manshouri R. Coyaud E. Kundu S. T. Peng D. H. Stratton S. A. Alton K.. (2019). ZEB1/NuRD complex suppresses TBC1D2b to stimulate E-cadherin internalization and promote metastasis in lung cancer. Nat. Commun. 10:5125. 10.1038/s41467-019-12832-z 31719531 PMC6851102

[B16] Mendonsa A. M. Na T. Y. Gumbiner B. M. (2018). E-cadherin in contact inhibition and cancer. Oncogene 37, 4769–4780. 10.1038/s41388-018-0304-2 29780167 PMC6119098

[B17] Miller J. J. Gaiddon C. Storr T. (2020). A balancing act: using small molecules for therapeutic intervention of the p53 pathway in cancer. Chem. Soc. Rev. 49, 6995–7014. 10.1039/D0CS00163E 32869798

[B18] Mircetic J. Dietrich A. Paszkowski-Rogacz M. Krause M. Buchholz F. (2017). Development of a genetic sensor that eliminates p53 deficient cells. Nat. Commun. 8:1463. 10.1038/s41467-017-01688-w 29133879 PMC5684360

[B19] Moreno A. M. Fu X. Zhu J. Katrekar D. Shih Y. V. Marlett J.. (2018). *In situ* gene therapy via AAV-CRISPR-Cas9-mediated targeted gene regulation. Mol. Ther. 26, 1818–1827. 10.1016/j.ymthe.2018.04.017 29754775 PMC6035733

[B20] Muller P. Y. Milton M. N. (2012). The determination and interpretation of the therapeutic index in drug development. Nat. Rev. Drug Discov. 11, 751–761. 10.1038/nrd3801 22935759

[B21] Nihongaki Y. Kawano F. Nakajima T. Sato M. (2015). Photoactivatable CRISPR-Cas9 for optogenetic genome editing. Nat. Biotechnol. 33, 755–760. 10.1038/nbt.3245 26076431

[B22] Onder T. T. Gupta P. B. Mani S. A. Yang J. Lander E. S. Weinberg R. A. (2008). Loss of E-cadherin promotes metastasis via multiple downstream transcriptional pathways. Cancer Res. 68, 3645–3654. 10.1158/0008-5472.CAN-07-2938 18483246

[B23] Polstein L. R. Gersbach C. A. (2015). A light-inducible CRISPR-Cas9 system for control of endogenous gene activation. Nat. Chem. Biol. 11, 198–200. 10.1038/nchembio.1753 25664691 PMC4412021

[B24] Qi L. S. Larson M. H. Gilbert L. A. Doudna J. A. Weissman J. S. Arkin A. P.. (2013). Repurposing CRISPR as an RNA-guided platform for sequence-specific control of gene expression. Cell 152, 1173–1183. 10.1016/j.cell.2013.02.022 23452860 PMC3664290

[B25] Ran F. A. Cong L. Yan W. X. Scott D. A. Gootenberg J. S. Kriz A. J.. (2015). *In vivo* genome editing using *Staphylococcus aureus* Cas9. Nature 520, 186–191. 10.1038/nature14299 25830891 PMC4393360

[B26] Rivière I. Sadelain M. (2017). Chimeric antigen receptors: a cell and gene therapy perspective. Mol. Ther. 25, 1117–1124. 10.1016/j.ymthe.2017.03.034 28456379 PMC5417838

[B27] Santiago-Ortiz J. L. Schaffer D. V. (2016). Adeno-associated virus (AAV) vectors in cancer gene therapy. J. Control. Release 240, 287–301. 10.1016/j.jconrel.2016.01.001 26796040 PMC4940329

[B28] Schenone M. Dančík V. Wagner B. K. Clemons P. A. (2013). Target identification and mechanism of action in chemical biology and drug discovery. Nat. Chem. Biol. 9, 232–240. 10.1038/nchembio.1199 23508189 PMC5543995

[B29] Sedlmayer F. Aubel D. Fussenegger M. (2018). Synthetic gene circuits for the detection, elimination and prevention of disease. Nat. Biomed. Eng. 2, 399–415. 10.1038/s41551-018-0215-0 31011195

[B30] Senís E. Fatouros C. Große S. Wiedtke E. Niopek D. Mueller A. K.. (2014). CRISPR/Cas9-mediated genome engineering: an adeno-associated viral (AAV) vector toolbox. Biotechnol. J. 9, 1402–1412. 10.1002/biot.201400046 25186301

[B31] Strumane K. Berx G. Van Roy F. (2004). Cadherins in cancer. Handb. Exp. Pharmacol. 2004, 69–103. 10.1007/978-3-540-68170-0_4 20455091

[B32] Truong D. J. Kühner K. Kühn R. Werfel S. Engelhardt S. Wurst W.. (2015). Development of an intein-mediated split-Cas9 system for gene therapy. Nucleic Acids Res. 43, 6450–6458. 10.1093/nar/gkv601 26082496 PMC4513872

[B33] Valente J. F. A. Queiroz J. A. Sousa F. (2018). p53 as the focus of gene therapy: past, present and future. Curr. Drug Targets 19, 1801–1817. 10.2174/1389450119666180115165447 29336259

[B34] van Roy F. Berx G. (2008). The cell-cell adhesion molecule E-cadherin. Cell. Mol. Life Sci. 65, 3756–3788. 10.1007/s00018-008-8281-1 18726070 PMC11131785

[B35] Vasekar M. Degraff D. Joshi M. (2016). Immunotherapy in bladder cancer. Curr. Mol. Pharmacol. 9, 242–251. 10.2174/1874467208666150716120945 26177642

[B36] Verdera H. C. Kuranda K. Mingozzi F. (2020). AAV vector immunogenicity in humans: a long journey to successful gene transfer. Mol. Ther. 28, 723–746. 10.1016/j.ymthe.2019.12.010 31972133 PMC7054726

[B37] Wong S. H. M. Fang C. M. Chuah L. H. Leong C. O. Ngai S. C. (2018). E-cadherin: its dysregulation in carcinogenesis and clinical implications. Crit. Rev. Oncol. Hematol. 121, 11–22. 10.1016/j.critrevonc.2017.11.010 29279096

[B38] Wright A. V. Sternberg S. H. Taylor D. W. Staahl B. T. Bardales J. A. Kornfeld J. E.. (2015). Rational design of a split-Cas9 enzyme complex. Proc. Natl. Acad. Sci. U.S.A. 112, 2984–2989. 10.1073/pnas.1501698112 25713377 PMC4364227

[B39] Wu Z. Yang H. Colosi P. (2010). Effect of genome size on AAV vector packaging. Mol. Ther. 18, 80–86. 10.1038/mt.2009.255 19904234 PMC2839202

[B40] Yamada M. Nagasaki S. C. Suzuki Y. Hirano Y. Imayoshi I. (2020). Optimization of light-inducible Gal4/UAS gene expression system in mammalian cells. iScience 23:101506. 10.1016/j.isci.2020.101506 32919371 PMC7491154

[B41] Ye H. Fussenegger M. (2019). Optogenetic medicine: synthetic therapeutic solutions precision-guided by light. Cold Spring Harb. Perspect. Med. 9:a034371. 10.1101/cshperspect.a034371 30291146 PMC6719591

[B42] Zarrin A. A. Malkin L. Fong I. Luk K. D. Ghose A. Berinstein N. L. (1999). Comparison of CMV, RSV, SV40 viral and Vlambda1 cellular promoters in B and T lymphoid and non-lymphoid cell lines. Biochim. Biophys. Acta 1446, 135–139. 10.1016/S0167-4781(99)00067-6 10395926

[B43] Zetsche B. Volz S. E. Zhang F. (2015). A split-Cas9 architecture for inducible genome editing and transcription modulation. Nat. Biotechnol. 33, 139–142. 10.1038/nbt.3149 25643054 PMC4503468

[B44] Zhan H. Xie H. Zhou Q. Liu Y. Huang W. (2018). Synthesizing a genetic sensor based on CRISPR-Cas9 for specifically killing p53-deficient cancer cells. ACS Synth. Biol. 7, 1798–1807. 10.1021/acssynbio.8b00202 29957992

[B45] Zhan T. Rindtorff N. Betge J. Ebert M. P. Boutros M. (2019). CRISPR/Cas9 for cancer research and therapy. Semin. Cancer Biol. 55, 106–119. 10.1016/j.semcancer.2018.04.001 29673923

[B46] Zhang F. Cong L. Lodato S. Kosuri S. Church G. M. Arlotta P. (2011). Efficient construction of sequence-specific TAL effectors for modulating mammalian transcription. Nat. Biotechnol. 29, 149–153. 10.1038/nbt.1775 21248753 PMC3084533

[B47] Zhao W. Wang Y. Liang F. S. (2020). Chemical and light inducible epigenome editing. Int. J. Mol. Sci. 21:998. 10.3390/ijms21030998 32028669 PMC7037166

[B48] Zhu H. J. Zhang Z. Q. Zeng X. F. Wei S. S. Zhang Z. W. Guo Y. L. (2004). Cloning and analysis of human UroplakinII promoter and its application for gene therapy in bladder cancer. Cancer Gene Ther. 11, 263–272. 10.1038/sj.cgt.7700672 14963492

