# Supplementary material for: A Light-Inducible Split-dCas9 System for Inhibiting the Progression of Bladder Cancer Cells by Activating p53 and E-cadherin
Source: Front Mol Biosci. 2021 Jan 5;7:627848. doi: 10.3389/fmolb.2020.627848 (PMC7814291; doi:10.3389/fmolb.2020.627848)
Supplement: Supplementary file 1 [file Table_1.DOCX]

**A light-inducible split-dcas9 system for inhibiting the progression of bladder cancer cells by activating p53 and E-cadherin**

Xinbo Huang et al.

**Supplementary Table.1 Maps of plasmids used in this study.**

| pHS-AVC-LW502 | pZDonor-CMV-NLS-CIBN-dcas9C(1-1153)-IntN-NLS |
| --- | --- |
| pHS-AVC-LW503 | pZDonor-CMV-NLS-IntC-dcas9C(1154-1368)-CIBN-NLS |
| pHS-AVC-LW504 | pZDonor-hU6-TRE sgRNA-sgRNA_backbone-CMV-NLSX3-CRY2PHR-NLS-VPR |
| pHS-AVC-LW549 | pZDonor-CMV-mkate-terminator-TRE-EYFP-SV40 |
| pHS-AVC-LW550 | pZD_Seq1-TRE-hluc-SV40 promoter-hRluc-Seq2 |
| pHS-AVC-LW550 | pZD_Seq1-TRE-p53 -SV40 promoter-hRluc |
| pHS-AVC-LW667 | pZDonor-hTERT-NLS-CIBN-dcas9N(1-1153)-IntN-NLS |
| pHS-AVC-LW668 | pZDonor-hUPII promoter-NLS-IntC-dcas9C(1154-1368)-CIBN-NLS |
| pHS-ACR-LW347 | pZDonor-hU6-CDH1 sgRNA1(human,chr16:68771036,CDH,TSS:-158)-sgRNA backbone-CMV-NLSX3-CRY2PHR-NLS-VPR |
| pHS-ACR-LW348 | pZDonor-hU6-CDH1 sgRNA2(human,chr16:68771000,CDH,TSS:-194)-sgRNA backbone-CMV-NLSX3-CRY2PHR-NLS-VPR |
| pHS-ACR-LW349 | pZDonor-hU6-CDH1 sgRNA3(human,chr16:68770992,CDH,TSS:-158)-sgRNA backbone-CMV-NLSX3-CRY2PHR-NLS-VPR |
| pHS-ACR-LW350 | pZDonor-hU6-Control sgRNA- sgRNA_backbone-CMV-NLSX3 -CRY2PHR-NLS-VPR |

**Supplementary Table.2 Relative sequences used in this study.**

| Name | cDNA sequence |
| --- | --- |
| hTERT promoter | GGCCCCTCCCTCGGGTTACCCCACAGCCTAGGCCGATTCGACCTCTCTCCGCTGGGGCCCTCGCTGGCGTCCCTGCACCCTGGGAGCGCGAGCGGCGCGCGGGCGGGGAAGCGCGGCCCAGACCCCCGGGTCCGCCCGGAGCAGCTGCGCTGTCGGGGCCAGGCCGGGCTCCCAGTGGATTCGCGGGCACAGACGCCCAGGACCGCGCTCCCCACGTGGCGGAGGGACTGGGGACCCGGGCACCCGTCCTGCCCCTTCACCTTCCGGCTCCGCCTCCTCCGCGCGGACCCCGCCCCGTCCCGACCCCTTCCGGGTTTCCGGCCCAGCCCCTTCCGGGCCCTCCCAGCCCCTCCCCTTCCTTTCCGGGGCCCCGCCCTCTCCTCGCGGCGCGAGTTTCCGGCAGCGCTGCGTCCTGCTGCGCACGTGGGAAGCCCTGGCCCCGGCCACCCCCGCG |
| hUPII promoter | GCTGGCAACTTCAAGTGTGGGCCTTTCAGACCGGCATCATCAGTGTTACGGGGAAGTCACTAGAAATGCAGAATTGATTGGGCACGGTGGCTCACACCTGTAATCCCAACACTCTGGGAGGCCAAGGCGGGTGGATCACTTGTGGTCAGGAGTTTGAGACCAGCCTGGCCAACATGGTGAAACCTCATCTCTACTAAAAATGCAAAAATTAGCTGGGAATGGTGGCACATGCCTATAATCCCAGTTACTCAGGAGGCTGAGGCAGGAGAATCGTTTGAACCTGGGAGGCAGAGGTTGCAGTGAGCCGAGATCACGCCACTGCACTCCAGCCTGGGTGACACAGCGAGACTCTGTCTCAAAAAAAAAAAAAAATGCAGAATTTCAGGCTTCACCCCAGACCCACTGCATGACTGCATGAGAAGCTGCATCTTAACAAGATCCCTGGTAATTCATACGCATATTAAATTTGGAGATGCACTGGCGTAAGACCCTCCTACTCTCTGCTTAGGCCCATGAGTTCTTCCTTTACTGTCATTCTCCACTCACCCCAAACTTTGAGCCTACCCTTCCCACCTTGGCGGTAAGGACACAACCTCCCTCACATTCCTACCAGGACCCTAAGCTTCCCTGGGACTGAGGAAGATAGAATAGTTCGTGGAGCAAACAGATATACAGCAACAGTCTCTGTACAGCTCTCAGGCTTCTGGAAGTTCTACAGCCTCTCCCGACAAAGTATTCCACTTTCCACAAGTAACTCTATGTGTCTGAGTCTCAGTTTCCACTTTTCTCTCTCTCTCTCTCTCTCAATTTTCTGAGACAGAGTTTCACTTAGTTGCCCAGGCTGGAGTGCAGGGGCACAATCTCGGCTCACTGCAACCTCCACCTCCTGGGTTCCAGTGTTTCTCCTGTCTCAGCCTCCCGAGTAGCTGGGATTACAGGCACACACCACCGCGTTAGTTTTTGTATTTTTGGTAGAGATGGTGTTTCGCCATATTGGCCAGGCTGATCTCGAACTCCTGACCTCAGGTGATCCGCCCACCTCGGCCTCCCAAAGTGCTGGGATTACAGGCATGAGCCACCACGCCCGGCTGATCTCTTTTCTATTTTAATAGAGATCAAACTCTCTGTGTTGCCTAGGCTGGTCTTGAACTCCTGGCCTCGAGTGATCCTCCCACCTTGGCCTCCCAAAGTGCTGGGATTACAGGCATGAGCCACTGTGCCTGGCCTCAGTTCTACTACAAAAGGAAGCCAGTACCAGCTACCACCCAGGGTGGCTGTAGGGCTACAATGGAGCACACAGAACCCCTACCCAGGGCCCGGAAGACGTCCTGACTCCTCTCCCCTCCCTCTGCTCAGAACTCCTCTGCTTCTTTCTGATGTAGCCCAGGGCTGGAGGAGGCAGTCAGGGAAGTTCTGTCTCTTTTTCATGTTATCTTACGAGGTCTCTTTTCTCCATTCTCAGTTCAACAAATGGTTGCTGCCCAAGGCTGACTGTGCCCACCCCCAACCCCTGCTGGCCAGGGTCAATGTCTGTCTCTCTGGTCTCTCCAGAAGTCTTCCATGGCCACCTTCGTCCCCACCCTCCAGAGGAATCTGAAACCGCATGTGCTCCCTGGCCCCCACAGCCCCTGCCTCTCCCAGAGCAGCAGTACCTAAGCCTCAGTGCACTCCAAGAATTGAAACCCTCAGTCTGCTGCCCCTCCCCACCAGAATGTTTCTCTCCCATTCTTACCCACTCAAGGCCCTTTCAGTAGCCCCTTGGAGTATTCTCTTCCTACATATCAGGGCAACTTCCAAACTCATCACCCTTCTGAGGGGTGGGGGAAAGACCCCCACCACATCGGGGGAGCAGTCCTCCAAGGACTGGCCAGTCTCCAGATGCCCGTGCACACAGGAACACTGCCTTATGCACGGGAGTCCCAGAAGAAGGGGTGATTTCTTTCCCCACCTTAGTTACACCATCAAGACCCAGCCAGGGCATCCCCCCTCCTGGCCTGAGGGCCAGCTCCCCATCCTGAAAAACCTGTCTGCTCTCCCCACCCCTTTGAGGCTATAGGGCCCAAGGGGCAGGTTGGACTGGATTCCCCTCCAGCCCCTCCCACCCCCAGGACAAAATCAGCCACCCCAGGGGCAGGGCCTCACTTGCCTCAGGAACCCCAGCCTGCCAGCACCTATTCCACCTCCCA |
| TRE sgRNA | TACGTTCTCTATCACTGATA |
| CDH1 sgRNA1 | CACCCGGCCTCGCATAGACG |
| CDH1 sgRNA2 | TGGAGTTGCTAGGGTCTAGG |
| CDH1 sgRNA3 | CTAGGGTCTAGGTGGGTTAT |

**
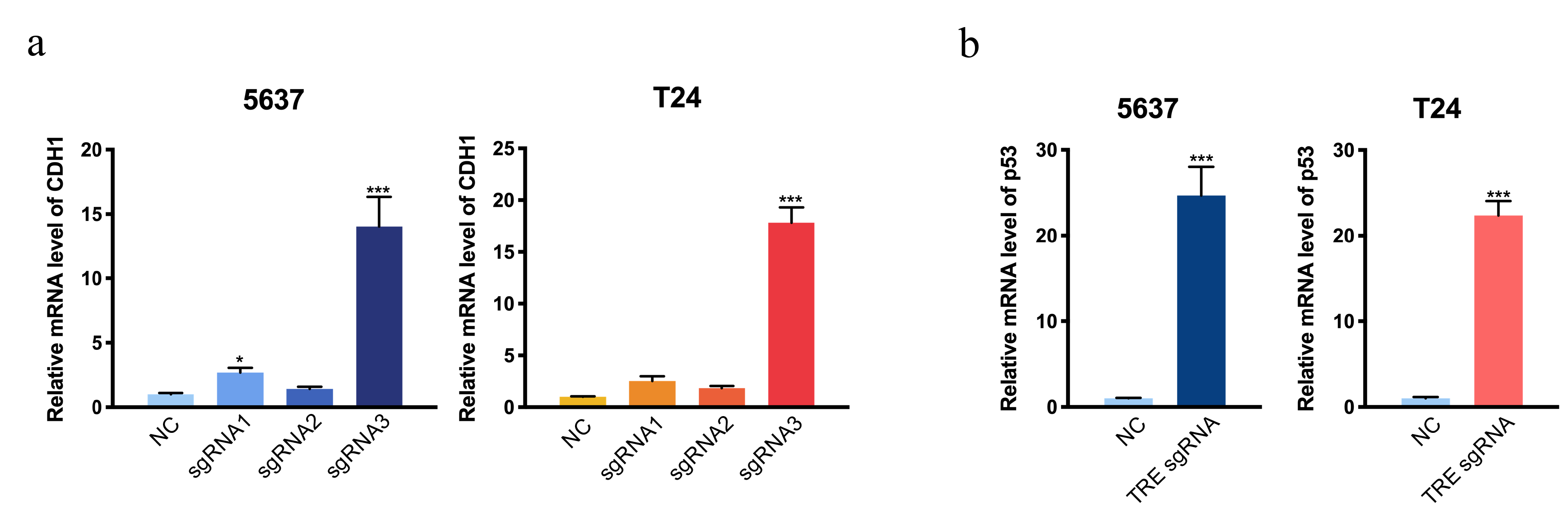
**

**Supplementary Figure.1 The expression level of p53 and CHD1 mRNA.**

**(a)** Effects of CHD1 sgRNAs (1, 2, 3) on the relative CDH1 mRNA level in 5637 and T24 cells, compared to the negative control. **(b)** Effect of TRE sgRNA on the relative p53 mRNA level in 5637 and T24 cells, compared to the negative control. Data are means ± SD. (n=3, **p* < 0.05, ***p* < 0.01, ****p* < 0.001)
